# The link between ancient microbial fluoride resistance mechanisms and bioengineering organofluorine degradation or synthesis

**DOI:** 10.1038/s41467-024-49018-1

**Published:** 2024-05-30

**Authors:** Randy B. Stockbridge, Lawrence P. Wackett

**Affiliations:** 1 Department of Molecular, Cellular, and Developmental Biology, University of Michigan, Ann Arbor, MI 48109 USA; 2 Department of Biochemistry, Biophysics & Molecular Biology and Biotechnology Institute, University of Minnesota, Minneapolis, MN 55455 USA

**Keywords:** Environmental biotechnology, Applied microbiology, Bioremediation, Metabolic engineering

## Abstract

Fluorinated organic chemicals, such as per- and polyfluorinated alkyl substances (PFAS) and fluorinated pesticides, are both broadly useful and unusually long-lived. To combat problems related to the accumulation of these compounds, microbial PFAS and organofluorine degradation and biosynthesis of less-fluorinated replacement chemicals are under intense study. Both efforts are undermined by the substantial toxicity of fluoride, an anion that powerfully inhibits metabolism. Microorganisms have contended with environmental mineral fluoride over evolutionary time, evolving a suite of detoxification mechanisms. In this perspective, we synthesize emerging ideas on microbial defluorination/fluorination and fluoride resistance mechanisms and identify best approaches for bioengineering new approaches for degrading and making organofluorine compounds.

## Abstract (web summary)

Microbial degradation and biosynthesis of fluorinated compounds is a field of increasing importance, but is hampered by the significant toxicity of fluoride. Here authors discuss emerging ideas on microbial defluorination/fluorination and fluoride resistance mechanisms, providing guidance on how this knowledge can guide future bioengineering approaches.

## Introduction

Early humans harnessed organic compounds, transition metals, and salts, but they largely avoided fluorine until the nineteenth century. At that time, chemists known as the “fluorine martyrs” experimented with fluorine gas and hydrogen fluoride, ultimately to the detriment of their health^1^. The first nucleophilic halogen exchange reaction to introduce fluorine into an organic molecule was carried out by the chemist and composer Aleksandr Borodin in 1862^2^. After Henri Moissan was awarded the 1906 Nobel Prize in Chemistry for methods to more safely handle fluorine, organofluorine chemistry blossomed. Another marked uptick in fluorine chemistry occurred in the 1940s and 1950s, as a direct consequence of the Manhattan Project and the need to enrich uranium as the hexafluoride, bringing the field to its current state^3^. Today thousands of fluorinated compounds have entered commerce^4,5^. For example, over the last 22 years, more than 50% of newly registered pesticides are organofluorine compounds^6^. Another prominent class of organofluorine compounds is designated as PFAS, standing for per- and poly-fluoroalkyl substances. We will use PFAS broadly here to refer to per- and polyfluorinated compounds, particularly those with -CF_2_- and -CF_3_ functionality.

Over the last ~80 years, PFAS has become widely used because of their high degree of chemical stability, lower boiling points than compounds of comparable mass, and propensity to form a fluorous phase characterized by immiscibility in water and typical organic solvents^7^. These properties make them ideal as heat exchange agents, fire-fighting foams, water repellents, non-stick agents, and chemically resistant polymers. Combining perfluorinated alkyl chains with polar groups such as carboxylates, sulfonates, or amines generates amphiphilic molecules that serve as detergents and surface active agents. While the precise number is constantly changing due to pressure for replacement, there are still more than one thousand PFAS compounds in current commercial use.

Research on PFAS distribution and toxicity has raised alarm about their environmental persistence^3^, their accumulation in blood serum and tissue^8^, and their physiological effects^9,10^, leading to stringent environmental regulations for certain PFAS^11^. Globally, almost the entire human population has detectable blood serum levels of PFAS^8^, in major part due to environmental exposure from water or food sources^12^. Organofluorine contamination has emerged as one of the most pressing environmental regulatory issues over the last five years, and it is expected that regulatory constraints will extend to more compounds, highlighting the necessity for PFAS and organofluorine remediation and replacement.

Currently, remediation and replacement efforts largely use physical and chemical methods^13,14^. However, increasingly urgent calls for mitigation worldwide have generated intense interest in finding novel, sustainable ways to reduce or minimize exposure to PFAS and organofluorine pollution^15–18^. It is within this context that the first microbial solutions to PFAS contamination are advancing. These fall into two major categories: (1) remediation of contaminants, for example, treatment of concentrated PFAS waste in bioreactors, or perhaps highly contaminated natural systems, and (2) biotechnology to develop high-performance organofluorine chemicals with a lower fluorine content than some current commercial PFAS. As an example of the growing interest in such biosynthetic approaches, a consortium funded by the European Commission, consisting of eight academic institutions, and industry and manufacturing entities, recently embarked on a major effort to develop fluorinated polymer precursors biologically in order to replace their chemical synthesis (https://www.sinfoniabiotec.eu). In addition, two other applications in the biosynthesis of fluorinated compounds are for generating fast-decaying ^18^F-isotope labeled molecules for Positron Emission Tomography (PET)^19^ and specifically fluorinated natural products such as antiviral agents^20^.

Microbial approaches to address organofluorine pollution have several advantages. Bioremediation would be cheaper and more effective than physico-chemical remediation in some circumstances if suitable biological systems can be identified or engineered. Likewise, biologically-generated replacement chemicals could be designed to have less toxicity and greater biodegradability. Enzymatic synthesis of fluorinated organic molecules has the potential to make tailored structures that are difficult to make via conventional synthesis^20^.

In this Perspective, we focus on the microbial physiology that underlies these promising biological approaches for solving problems related to organofluorine persistence. In particular, organofluorine degradation yields fluoride ions, and biological syntheses use NaF. Though NaF is much safer for humans than HF and fluorine gas, fluoride is highly toxic to the bacteria carrying out biosynthetic or biodegradation reactions^21,22^. We will review how these applications have been thwarted by the toxicity of fluoride for bacteria, and present a perspective on how this problem might be overcome. We argue that applying fundamental physiological considerations to bioengineer solutions to PFAS and organofluorine persistence can help circumvent some of the common roadblocks that have emerged in these fields of study.

## An overview of microbial organofluorine metabolism

### Naturally evolved fluorinated natural product metabolism

**Fig. 1:**
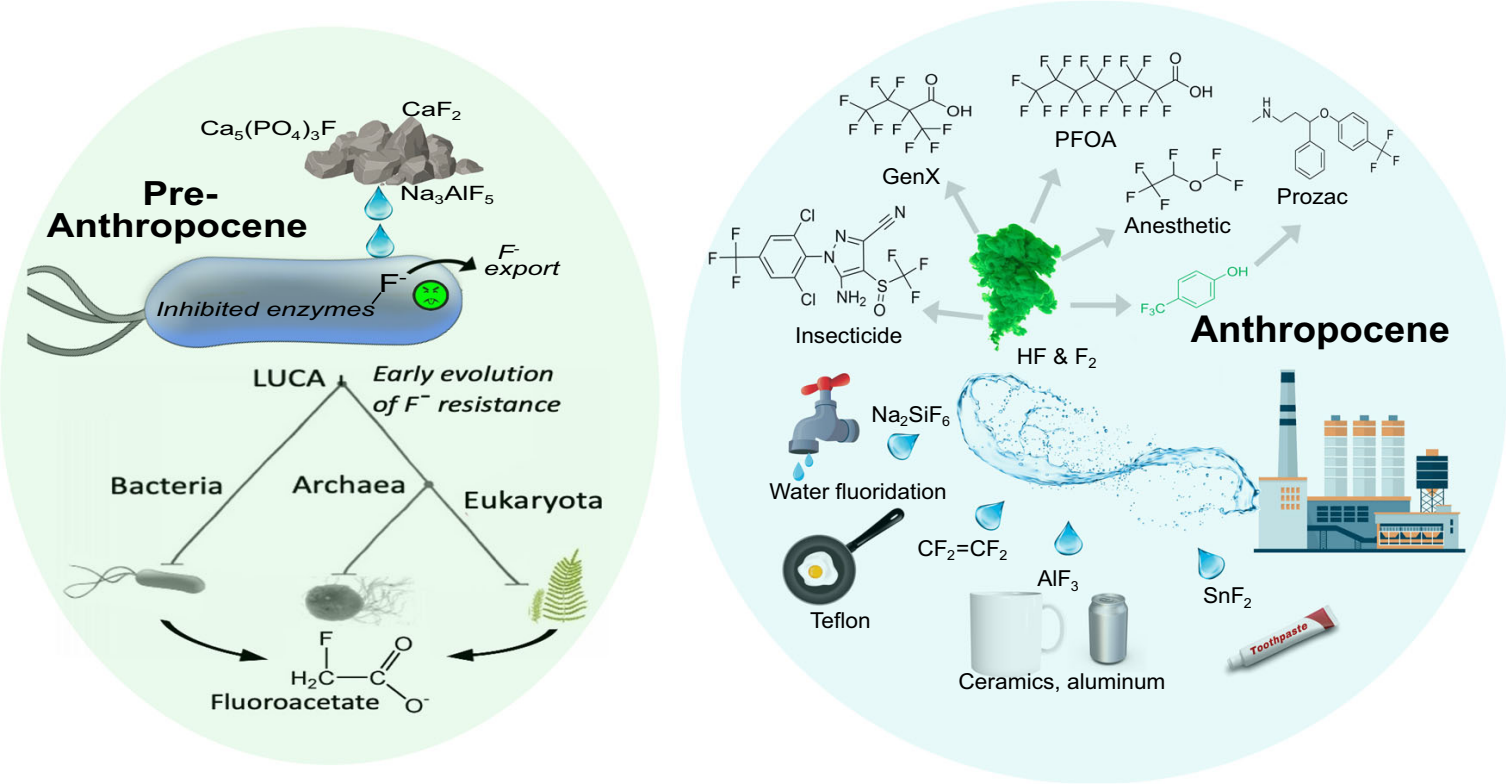
As the 13th most abundant element in Earth’s crust, fluorine has interacted with living systems since the inception of cellular life. Left, During the pre-Anthropocene era of life, covering ~3.8 billion years, fluorine in the form of fluoride anion (F^−^), derived largely from minerals, exhibited toxicity to cells and protocells by binding to Mg^2+^ and Ca^2+^ centers in enzymes or ribozymes. Fluoride export functions arose early in evolution. Today, most living things avoid fluorine, but a few rare plants and prokaryotes naturally evolved to biosynthesize fluoroacetate as a metabolic toxin to kill competitors and predators. Right, In the last 100 years and into the Anthropocene, humans have exposed the biosphere to a tsunami of inorganic and organic fluorine compounds. Of greatest concern are the large number of per- and polyfluorinated compounds (PFAS), such as perfluorooctanoic acid (PFOA) and 2,3,3,3-tetrafluoro-2-(heptafluoropropoxy)propanoic acid (GenX), shown at the top right. PFAS are persistent in the environment, raising human and ecosystem health concerns.

Fluorine is more common in the earth’s crust than phosphorus, nitrogen, and sulfur and yet a survey of the elemental composition of several dozen prokaryotes did not identify fluorine amongst the 33 elements found, making it less prevalent than non-biological metals like cadmium, tin, and silver^23^. Although most microbes and plants have evolved to minimize fluorine assimilation, very few have harnessed fluorochemistry (Fig. 1). Plants of several varieties in Australia, Africa, and South America produce fluoroacetate to deter feeding by animals, since the compound becomes highly toxic to central metabolism after metabolic conversion to fluorocitrate^24,25^. Fluoroacetate production is thought to be an ancient function given the geographic and taxonomic distribution of the fluoroacetate producers. Even fewer organisms make other monofluorinated natural products, for example, 4-fluoro-L-threonine^26^ or fluorinated fatty acids^27^, which are also toxic metabolite mimics, and the fluorinated antibiotic nucleocidin^28^.

### Enzymatic degradation of organofluorines

Naturally evolved defluorinase enzymes are inherently rare given the scarcity of fluorinated natural products, such as fluoroacetate described above^29^. The early discovery of a bacterial fluoroacetate dehalogenase belonging to the α/β-hydrolase superfamily^30^ spurred additional discovery efforts for fluoroacetate dehalogenase activity within this family^31^. More recently, members of the haloacid dehalogenase-like hydrolase (HAD) superfamily have also been shown to catalyze the defluorination of fluoroacetate to yield glycolic acid and HF^32^. These fluoroacetate dehalogenases prefer fluorine over other halogen substituents, suggesting that they were selected for fluoroacetate detoxification or assimilation of fluoroacetate as a carbon source^30,33^. The X-ray structure of fluoroacetate dehalogenase from *Rhodopseudomonas palustris* revealed a highly specific and compact binding site for the fluorine atom of the substrate, which is suggested to significantly enhance catalytic C–F bond cleavage^34^.

In most cases, canonical fluoroacetate dehalogenases exhibit little to no activity with α,α-difluoro carboxylic acids characteristic of many PFAS chemicals of interest to remediate. However, several defluorinases that act on difluoroacetic acid have been discovered recently, holding open the possibility that naturally evolved enzymes might be able to degrade longer chain α,α-difluoro acids^35^. The enzyme products of difluoroacetic acid defluorination are two fluoride anions and an α-keto acid. Since α-keto acid decarboxylases are known, a plausible degradative pathway for perfluorocarboxylic acids would use consecutive paired reactions of α,α-defluorination and decarboxylation, releasing fluoride ions at each step and culminating in trifluoroacetic acid, an end product that is highly recalcitrant to further degradation.

Other enzymes with defluorination activity have also been identified, although it is less clear that these evolved naturally for the purpose of C–F bond cleavage. For example, 2 or 4-fluorobenzoate serves as a substrate for the anaerobic growth of bacteria that also grow on the common natural substrate benzoate^36^. Defluorination during 4-fluorobenzoate metabolism was shown to occur following ring reduction, catalyzed by the enoyl-CoA hydratase/hydrolase that participates in benzoate assimilation^37^. However, in these cases, defluorination is considered to be a promiscuous enzyme activity^38^. Likewise, a number of oxygenases have been demonstrated to support growth on fluorinated alkanes and aromatics in addition to naturally abundant substrates^38,39^. In other cases, oxygenases have been shown to participate in a non-growth dependent, promiscuous release of fluorine from fluorinated alkanes^40^, alkenes^41^, and aromatics^42,43^. The reactions typically proceed through the formation of *gem*-fluoro alcohols that undergo spontaneous elimination of HF.

Biological systems that degrade perfluorinated compounds have also been reported recently^44,45^, including reports of the defluorination of perfluorinated acids in consortia^46–48^ and by a single bacterium^49^. While no enzymes were directly identified in those studies, the reactions are proposed to be reductive based on the identity of the products and gene expression studies^50^. Most recently, the electron bifurcating caffeoyl-CoA reductase system of *Acetobacterium* spp. was implicated in the reductive defluorination of perfluorinated unsaturated carboxylic acids^51^.

### Bioengineering organofluorine synthesis to replace PFAS

With more than 200 applications and thousands of individual chemicals in commercial use^7^, PFAS utilization will continue for the foreseeable future. However, there are intense efforts underway in industry and academia to replace the most problematic compounds with more lightly fluorinated analogs^20–22^. Current fluorination reactions often use HF or other hazardous reagents, and regio-selective synthesis of partially fluorinated compounds is difficult^52^. Many fluorinating reagents used in organic synthesis are toxic and highly unstable in water. There is a need for better control and safety in organofluorine synthesis. In this context, an emerging area of organofluorine synthesis research is on biosynthetic approaches that use enzymes to carry out fluorination chemistry and perform reactions in aqueous solutions.

It would be ideal to prepare specific fluorinated molecules using simple fluoride salts, such as NaF or KF. Nature offers the enzyme fluorinase, which catalyzes the reaction of fluoride anion and S-adenosyl-L-methionine (SAM) to make 5′-fluoro-5′deoxyadenosine (5′-FDA) and L-methionine^53^. Initially identified in the fluoroacetate-producing bacterium *Streptomyces cattleya*, the fluorinase reaction thus uses fluoride ion to make a C–F bond and is the main entry point for fluorine in the production of the natural products fluoroacetaldehyde, fluoroacetate, and 4-fluoro-L-threonine^54^. However, fluorinase also has limitations for biosynthesis, including low *k*_cat_ values (up to 0.4 min^−1^)^55^ and high *K*_m_ values for fluoride (2 mM for the well-studied *S. cattleya* enzyme)^20^. Fluoride is a poor nucleophile in water due to the high free energy of hydration of the anion^56^, which must be overcome by the enzyme to catalyze a nucleophilic displacement reaction with SAM. A range of other fluorinase enzymes have been tested but so far, none show markedly different steady-state kinetic parameters^55^. Likewise, mutagenesis strategies have not significantly lowered the K_m_ for fluoride^57^. Nonetheless, fluorinase has been successfully leveraged in biosynthetic pathways to produce a variety of simple organofluorines used as PET tracers and medical imaging agents^58–60^. Moreover, although fluorinases are rare, recent research has uncovered new and more diverse enzymes than previously known^55^. It is likely that ongoing research into the structure/function relationships of these enzymes will continue to support advances in fluorine biocatalysis in the near future. In addition, there is evidence that other types of enzymes can act as entry points for fluorine into organic molecules, for example, those that contribute to the synthesis of omega-fluoro oleic acid^61^, which presumably originates from fluoroacetyl-CoA, or the fluorinated nucleoside antibiotic nucleocidin^62^. Additional research on these rare enzymatic chemistries will be essential to advance the biosynthesis of PFAS replacement chemicals.

## Fluoride stress is a key challenge for organofluorine metabolism

Earlier studies on biological organofluorine synthesis and degradation strongly indicate that fluoride anion toxicity is a major impediment to progress in both fields of study^21,55,63,64^. Unlike other monovalent anions like chloride, fluoride inhibits essential enzymes in central metabolism, often by forming tight complexes with metallo-cofactors like calcium or magnesium within the enzyme’s active site^65^. The fluoride inhibitory constants (*K*_i_ values) for enzymes like enolase (which catalyzes the penultimate step in glycolysis), pyrophosphatase, and various kinases are typically in the range of 100 μM^66^. Congruent with this, WT bacteria mount a gene expression response at intracellular fluoride concentrations around 60 μM^67^, and extracellular concentrations as low as 200 μM^68^.

Fluoride toxicity becomes particularly acute in the context of organofluorine bioremediation and biosynthesis. While studies that establish microbial degradation of PFAS are still in the early days, many research groups are searching for PFAS-degrading microorganisms, since microbial processes are more easily applied in scenarios with dilute PFAS that preclude technologies that rely on PFAS capture and concentration. Some commercial PFAS molecules contain 15 or more fluorines, and due to this atom’s electronegativity, C–F bond cleavage by any mechanism releases fluorine as a fluoride anion. Because fluoride cannot passively diffuse across the membrane, if this reaction occurs in the cytoplasm, fluoride will accumulate intracellularly. The intracellular volume of a typical bacterial cell is on the order of a femtoliter^69^ – in other words, if one femtomole of PFAS enters a cell and undergoes one C–F bond cleavage, the fluoride concentration will approach 1 M. In a hypothetical scenario of environmental PFAS degradation, a cell will approach toxic levels of intracellular fluoride (>100 μM) upon uptake and defluorination of perfluorooctanoic acid from just 10 pL of medium contaminated with 1 ppb of this substance, a value that is observed in PFAS-polluted environmental samples^70^. We have measured enzymatic C–F bond cleavage in one instance to be as fast as 10 bonds cleaved per second per enzyme (and there are thousands of enzymes per cell). Thus, for bacteria metabolizing such polyfluorinated compounds, PFAS or organofluorine degradation will rapidly release enough fluoride to impact cellular physiology. Moreover, fluoride produced intracellularly and released, or produced in the periplasm, readily re-enters and accumulates in cells via a process called weak acid accumulation^65,71^, especially as the medium pH acidifies, which is typical with dense bacterial cultures. For cultures of *P. putida* engineered to grow on fluorinated substrates, fluoride accumulates in the medium to concentrations exceeding 50 mM^63^. Similarly, the synthesis of PFAS alternatives by bacteria involves substantial fluoride stress, since the microbe must take up fluoride in a controlled manner as a substrate for synthesis. The mM K_m_ values of fluorinase for fluoride require that cells tolerate intracellular fluoride levels 100-fold greater than those that begin to elicit fluoride stress responses.

Observations from naturally evolved and engineered microbes highlight the biological challenges inherent to fluoride use. In *E. coli*, high concentrations of fluoride stall cell division and growth, which is only resumed after fluoride is removed^71^. Organisms that have evolved natural fluorination capabilities, like *S. cattleya*, separate growth and energy metabolism from organofluorine biosynthesis, only expressing fluorinases in stationary phase^72,73^. In an engineered strain of *Pseudomonas putida* expressing a defluorinase enzyme and grown on α-fluorocarboxylic acids, fluoride stress limits the growth rate^63^. Similarly, for *Acetobacterium* spp. that catalyze reductive defluorination of perfluorinated unsaturated carboxylic acids, strains that lacked a functional fluoride export protein failed to perform defluorination, linking fluoride detoxification and enzymatic defluorination^51^.

Biosynthetic reactions require circumventing bacteria’s fluoride defenses to deliver fluoride in a controlled manner. For example, biosynthesis of fluoroacetate in *E. coli* requires not only the biosynthetic genes from *S. cattleya*, but also chromosomal knockouts of the *crcB* gene, which encodes a fluoride channel^64^. Likewise, fluoroacetate synthesis in *P. putida* KT2440 was achieved by harnessing natural fluoride response elements to deliver fluoride to the cells in a controlled manner^55^.

## Microbial fluoride stress responses: recent advances

**Fig. 2:**
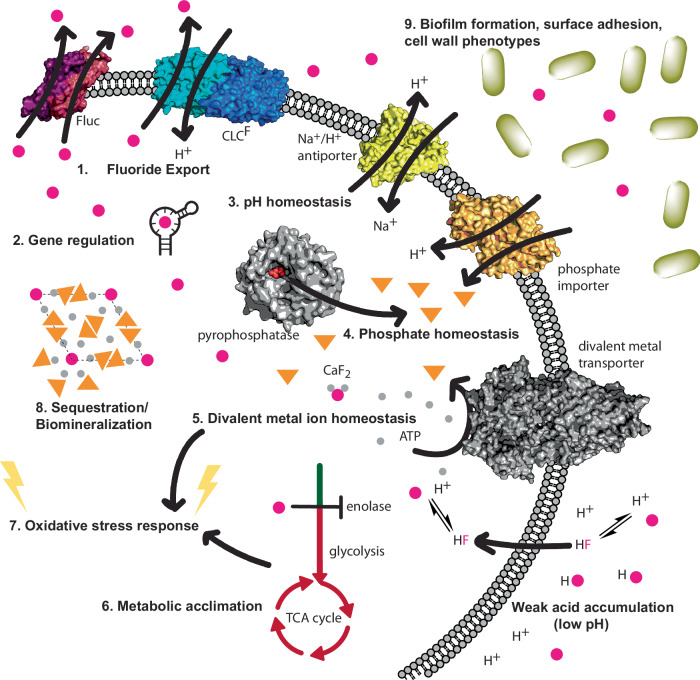
Microbial responses to fluoride stress. Pink spheres represent F^−^, orange triangles represent PO_4_^−^, and gray spheres represent Ca^2+^. Most bacteria exhibit a subset of these responses. (1) Flouride export is the first line of defense against environmental fluoride, which usually enters the cell via weak acid accumulation at low pH (bottom right). Bacteria typically encode one of the two types of fluoride exporters: Fluc (*crcB*) or CLC^F^. (2) Fluoride-responsive riboswitches are widespread among bacteria, upregulating the expression of genes involved in fluoride resistance. These bind fluoride as a Mg^2+^-fluoride complex. Other unknown mechanisms of gene regulation also exist. (3) Weak acid accumulation of fluoride reduces the proton-motive force and decreases the cytoplasmic pH, which cells counteract by expressing Na^+^/H^+^ antiporters. Fluoride-acclimated microbes exhibit enduring changes in pH homeostasis. (4) Various microbes overexpress inorganic pyrophosphatase, other phosphatases, and phosphate importers. This might be partly to surmount inhibition of phosphoryl transfer enzymes by fluoride, but it has also been shown that phosphate protects cells from fluoride stress. (5) Fluoride and divalent cations like Ca^2+^ and Mg^2+^ form poorly soluble complexes, which alters divalent metal ion homeostasis. Divalent cation transporters are overrepresented in operons with fluoride export proteins. (6) Fluoride inhibits several glycolytic enzymes, notably enolase, decreasing intermediates in lower glycolysis and the TCA cycle. Bacteria respond to this inhibition in various ways, including overexpression of glycolytic enzymes, metabolic shift to anaerobic fermentation, or pausing metabolism and growth. (7) As a consequence of the perturbations to oxidative metabolism and metal ion homeostasis, many microbes mount an oxidative stress response when fluoride levels are high. (8) Although less well understood as part of a natural fluoride response, some bacteria are able to synthesize minerals, such as fluorapatite (shown), with lattices that incorporate fluoride and effectively sequester this ion, intra- or extracellularly. (9) Many microbes exhibit changes in extracellular phenotypes like adhesion, biofilm formation, cell membrane structure and integrity, and polysaccharide export upon fluoride stress.

Although perfluorinated organic compounds are an anthropogenic stressor, fluoride resistance mechanisms are ancient and diverse (Fig. 2). Fluorine in the earth’s crust is largely found as fluoride bound in mineral form, for example: fluorite, fluorapatite, topaz, cryolite, sellaite, and villiaumite^74^. Fluorite (CaF_2_) is a mineral mined for fluorine extraction to make PFAS and other industrial fluorine-containing compounds. In acidic waters, in particular, fluoride can be extracted from minerals. Soil, seawater, and surface water typically possess fluoride concentrations in the tens-to-hundreds of micromolar concentration range^75^, which is high enough to elicit a biological response^67^. Thus, biological systems have been exposed to toxic levels of the anion over evolutionary time.

Environmental fluoride enters cells largely as HF^71^. With a pK_a_ value of 3.4, appreciable HF is formed in niches with a pH below ~7. HF is membrane permeant but dissociates to H^+^ and F^−^ at the physiological cytoplasmic pH. The ionic form becomes trapped in the cell, accumulating to levels dictated by the pH gradient across the membrane^71^. Via this process, even low levels of environmental fluoride can breach the cell and evoke a cellular stress response. Our understanding of these responses, which include fluoride export, modulation of pH and ion homeostasis, and metabolic rewiring, continue to advance (Fig. 2). We argue that by exploiting fundamental microbial physiologies — in particular fluoride stress responses — we can unlock better strategies for biodegradation or biosynthesis of organofluorine molecules such as PFAS. Although this review focuses on bacteria, fluoride stress responses are ancient and conserved, at least in part, among microorganisms. Thus, physiological fluoride responses in yeast and fungi will also be examined.

### Fluoride export

**Fig. 3:**
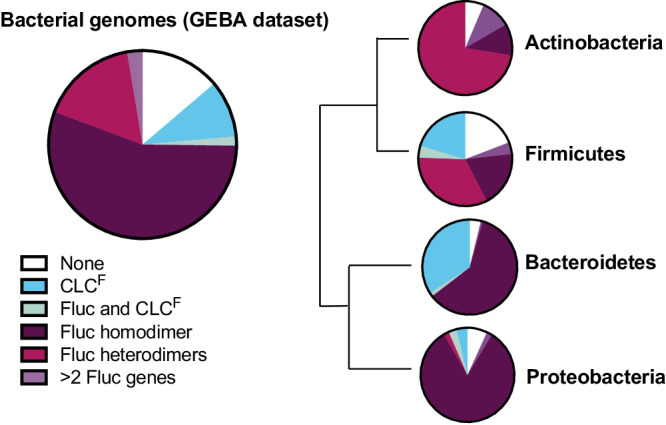
Distribution of fluoride exporters among representative bacterial species. Genomes are from a phylogenetically representative genomes set curated by the Joint Genome Institute (GEBA dataset, Genomic Encyclopedia of Bacteria and Archaea, bacterial genomes only)^76^. At right are the exporter distributions for four major bacterial phyla, with phylogenetic branch lengths according to ref. ^134^.

Across the tree of life, the first lines of defense against fluoride toxicity are membrane exporters that maintain this anion at low cytoplasmic concentrations^65^. Among bacteria, two fundamentally different fluoride export proteins have been identified, the CLC^F^s (gene name may be annotated *sycA*, *eriC*, *clcA*, *clcB*) and the Flucs (gene name may be annotated *crcB or fluC*)^65^. A survey of bacterial genomes from the Joint Genome Institute’s GEBA set of representative prokaryotic genomes^76^ shows that >85% of strains in the collection possess a fluoride exporter (Fig. 3). Species that lack fluoride exporters tend to be obligate intracellular symbionts with reduced genomes, for example members of the genera *Tenericutes, Spirochetes*, and *Fusobacteria*. The widespread distribution of these fluoride export genes among microbes emphasizes the pervasive impact of environmental fluoride over evolutionary time. The CLC^F^ and Fluc proteins are usually mutually exclusive in bacterial genomes; only ~3% of strains surveyed possess both. The importance of these exporters to fluoride resistance has been demonstrated in diverse organisms (Table 1).

**Table 1:** Fluoride resistance phenotypes and transport rates of fluoride export proteins

Species	Exporter Type	Genetic Knockout	Transport rate (ions/s)	Structure (PDB)
*Escherichia coli*	Fluc homodimer	MIC WT: 200 mM^67^ MIC KO: ~1 mM^67^		
*Streptococcus mutans*	CLC^F^	IC_50_ WT: ~150 mM^132^ IC_50_ KO: ~0.2 mM^68^	810^68^	
*Streptococcus sanguinus*	Fluc heterodimer	IC_50_ WT: ~300 mM^132^ KO: no growth at 75 mM^132^		
*Streptococcus anginosus*	CLC^F^	IC_50_ WT: ~300 mM^132^ IC_50_ KO: no growth at 75 mM^132^		
*Aspergillus fumigata*	FEX	MIC WT: 100 mM^111^ MIC KO: 1.56 mM^111^		
*Pseudomonas putida KT2440*	Fluc homodimer	IC_50_ WT: ~50 mM^21^ IC_50_ KO: ~1 mM^21^		
*Bordetella pertussis*	Fluc homodimer		>10^5^ ^81^	5A40^83^
*Enterococcus casseliflavus*	CLC^F^		880^77,78^	6D0J^79^
*Escherichia coli virulence plasmid*	Fluc homodimer		>10^5^ ^81^	5A43^83^
*Lactobacillus acidophilus*	Fluc heterodimer		>10^5^ ^81^	
*Piruella staleyi*	CLC^F^		810^77,78^	
*Candida albicans*	FEX	IC_50_ WT: 140 mM^85^ IC_50_ KO: .098 mM^85^		
*Sacchaomyces cervisiae*	FEX	IC_50_ WT: 70 mM^85^ IC_50_ KO: .06 mM^85^	>10^5 133^	
*Arabidopsis thailana*	FEX	WT MIC (seed germination): 4 mM^133^ KO MIC (seed germination): 0.1 mM^133^	>10^5^ ^133^	

The CLC^F^s are fluoride/proton antiporters that harness the proton gradient to expel fluoride from the cytoplasm^77^. These proteins are members of the large CLC (“**c**h**l**oride **c**hannel”) family of anion transporters and channels, which are found in all kingdoms of life. Biochemical experiments have demonstrated that CLC^F^s are >80-fold selective for fluoride over chloride, the halide that is closest in size and the main biological anion^77,78^. Fluoride-specific CLC^F^s can be identified based on three signature sequences: the GNNLI/GMGLI in the N-terminal domain that defines ion selectivity, GREGT/V at the heart of the transport machinery, and the GEVTP sequence in the C-terminal domain that contributes fluoride-binding residues^77–79^. Among the major bacterial phyla, CLC^F^s are found most frequently in *Bacteriodetes* (~30% of species) and *Firmicutes* (~20% of species). Many bacteria also possess additional CLC homologs for chloride transport; these are not capable of transporting fluoride^80^.

In contrast to the CLC^F^s, the Flucs (**Flu**oride **c**hannel) function as passive fluoride channels^81^ that exploit the positive-outside membrane potential (the electrical component of the proton-motive force) to drive the expulsion of this anionic species^71^. The Flucs are notable for their rapid rate of F^−^ efflux (10^6^ ions/s) and extremely high selectivity for fluoride over other anions, which exceeds 10,000-fold^81,82^. The Flucs are the most selective ion channel yet described, and no role besides fluoride export has been ascribed to any protein in this family^65^. The Flucs assemble as unusual antiparallel dimers, where one protomer is inserted into the membrane facing out, and the other is inserted into the membrane facing in^81,83^. Fluc proteins can be encoded by single genes, in which case a single protomer is inserted into the membrane in both inward and outward-facing conformations, assembling as an antiparallel homodimer, or they can be encoded by paired genes that express to form heterodimers of obligate inward- and outward-facing subunits^84^. Typically, the genes for the heterodimeric Flucs are adjacent to each other in genomes, and both genes are required for fluoride efflux activity^84^. Heterodimers are more common than homodimers among Gram-positive bacteria. Heterodimers are considerably rarer in Gram-negative phyla, which primarily express Flucs as homodimers.

Yeast, fungi, plants, and some ocean-dwelling animals like corals and sponges also possess fluoride exporters that belong to a third molecular family known as FEX^85,86^. FEX proteins are related structurally to the Flucs but possess a more complex two-domain fold^83,87^. Like the Flucs, these proteins export fluoride via a channel mechanism driven by the membrane potential^87^.

Although the fluoride exporters contribute most to fluoride resistance, different organisms still exhibit substantial variability in the fluoride concentrations that they tolerate (Table 1), suggesting that there are additional physiological determinants of fluoride resistance. In one illustration of the potential contribution of these additional fluoride resistance mechanisms, fluoride-acclimated strains of *Saccharomyces cerevisiae* exhibited a 700-fold gain in fluoride resistance, independent of fluoride export^88^.

### Fluoride-responsive gene regulation

Many bacteria exhibit a multilayered response to fluoride ions that is controlled at a genetic level. The best-studied regulatory element is the fluoride riboswitch, which binds fluoride as a fluoride-magnesium complex and upregulates the transcription of downstream genes^67,89^. Indeed, extensive riboswitch-based regulation is used by *S. cattleya* and *Methylobacterium DM4*, microbes capable of synthesizing and degrading fluorinated compounds, respectively^72,90^. As detailed below, a number of genes associated with microbial fluoride resistance are controlled by riboswitches. The most common proteins found in operons with these riboswitches are the fluoride exporters^67^, although only ~15% of Flucs and ~30% of CLC^F^s are regulated by riboswitches. While some fluoride response genes are constitutively expressed^71^, other unknown regulatory mechanisms also exist. For instance, in *Streptococcus mutans*, which do not possess a fluoride riboswitch, CLC^F^ expression is induced by fluoride addition to the medium^68^. A fluoride-resistant strain of *S. mutans* exhibits a constitutive expression of the CLC^F^ genes, and a single nucleotide polymorphism in the intergenic region 5′ to the operon was linked to this mechanism^91^.

### pH and ion homeostasis

Fluoride stress and pH homeostasis are intimately linked. Since membrane permeation of the weak acid HF is the major route to fluoride accumulation in the cytosol^92^, fluoride exposure is correlated with cytoplasmic acidification^21^. In addition, fluoride export is dependent on the proton-motive force – either as an explicit chemical driving force for the CLC^F^s, or as a component of the electrical gradient, as for the Flucs. As a result, maintaining pH homeostasis is important for fluoride resistance in microorganisms, and acid stress-response pathways are commonly upregulated in response to fluoride^88,93^. Indeed, after fluoride exporters, the next-most common class of riboswitch-associated genes is Na^+^/H^+^ antiporters^67^, which contribute to pH homeostasis in bacteria^94^. In fluoride-acclimated *S. cerevisiae*, one of the most frequent physiological adjustments was tolerance to lower homeostatic pH^88^.

There is also evidence that phosphate homeostasis is linked to fluoride resistance. The gene encoding pyrophosphatase is among the genes most frequently associated with fluoride riboswitches^67^, and in various organisms, including *Enterobacter cloacae* FRM^95^, *Acidithiophilus ferredoxins*^96^, and *S. cerevisiae*^97^, the expression of pyrophosphatase, polyphosphatase, and phosphate importers has been functionally linked to fluoride resistance. In addition, genes annotated as haloacid dehalogenases are often associated with riboswitches^67^. These were initially assumed to be related to fluorine metabolism, but it has since been shown that the majority of enzymes with the haloacid dehalogenase fold are in fact phosphoesterases^31,98^. This association between fluoride exposure and phosphatase upregulation might reflect overexpression to counteract inhibition of phosphoryl transfer enzymes by fluoride^99^. But there is also evidence that increased cytoplasmic phosphate is protective against fluoride toxicity^88,97^, perhaps partly due to phosphate’s buffering capacity at neutral pH.

In addition to phosphate importers and Na^+^/H^+^ antiporters, genes encoding other ion transporters are also overrepresented in fluoride-related operons and gene expression analyses^95^. Fluoride chelation impacts ion homeostasis, especially for divalent cations, and these perturbations can contribute to oxidative stress. The fluoride-related expression of ion transporters might thus be a response to fluoride-induced changes to divalent metal availability.

### Metabolic acclimation to fluoride

Fluoride-stressed microorganisms enact substantial, but sometimes dissimilar, changes to metabolism. While fluoride exerts broad-spectrum inhibition on any enzyme that relies on metal-ATP complexes, the enzymes of glycolysis are particularly sensitive. Enolase (which converts 2-phosphoglycerate to phosphoenolpyruvate, or PEP) is inhibited by fluoride with a *K*_i_ value of ~80 μM^100^. In *P. putida*, this is reflected by the accumulation of metabolites in upper glycolysis, and the depletion of PEP and other downstream products, including TCA cycle intermediates^21^. For some bacteria, the response to enolase inhibition by fluoride is to simply make more of the enzyme. Enolase is often observed in operons controlled by fluoride riboswitches^67^, and in fluoride-resistant *E. cloacae* FRM, enolase transcripts are upregulated 176-fold in response to fluoride^95^. Other bacteria, like *E. coli*, appear to shut down metabolism entirely, only resuming growth once the fluoride insult is removed^71^. For other bacteria, like *S. mutans*, there is evidence of a shift away from oxidative metabolism^101^. Formate hydrogen lyase, which oxidizes formic acid to produce ATP during anaerobic sugar fermentation, is another of the most common fluoride riboswitch-associated enzymes^67^. Fluoride-adapted *S. cerevisiae* also exhibit metabolic shifts to anaerobic fermentation pathways, and like *P. putida*, are depleted in TCA cycle intermediates^88^.

Correlations between amino acid pools and fluoride resistance have also been observed. For example, fluoride-stressed *P. putida* has elevated levels of methionine and tyrosine^21^. The gene encoding chorismate mutase, a key enzyme in the biosynthesis of aromatic amino acids, is one of the most common genes in fluoride exporter gene neighborhoods^67^, and its expression is upregulated in fluoride-resistant *S. mutans* together with the fluoride exporters in the same operon^91^. In this same fluoride-resistant *S. mutans* strain, pyruvate kinase, the enzyme that directs PEP towards pyruvate oxidation and the TCA cycle, is heavily mutated^91^, suggesting that other PEP-consuming biosynthetic pathways, such as aromatic amino acid production, might be favored under fluoride stress.

### Oxidative stress responses

Stress-response pathways, especially oxidative stress responses, are activated in a broad cross-section of bacterial species upon fluoride challenge^21,88,95,97,101^. Oxidative stress is associated with a number of the primary effects caused by fluoride described above, including intracellular acidification and disruption to the membrane potential, disruption of divalent metal homeostasis, and arrest of oxidative metabolism. Response to oxidative stress might also rationalize the association between aromatic amino acids and fluoride resistance, as tyrosine has been proposed to mitigate oxidative stress^102–104^. In yeast, fluoride resistance is imparted by the export of compounds like nitrates that contribute to oxidative stress, and the import of antioxidant metabolites like sulfite^97^.

### Cellular architecture, adhesion, and biofilm formation

In addition to the homeostatic and metabolic responses described above, fluoride exposure is also associated with changes to extracellular phenotypes. Genes associated with adhesion, biofilm formation, cell membrane structure and integrity, and polysaccharide export are upregulated in response to fluoride by diverse bacteria, including *P. putida*, *S. mutans*, *S. sobrinus*, and *A. ferrooxidans*^21,96,101^. Among the oral streptococci, fluoride has been shown to inhibit lectins that shape biofilm architecture^105^, and increase turnover of cell wall peptidoglycans, contributing to cellular lysis^106^. Similarly, changes to cell morphology, including cell shortening or lack of separation following septation, and increased extracellular carbohydrate content, are also caused by fluoride stress^63,107,108^. The influence of fluoride on cellular adhesive properties is also observed for eukaryotic microbes. Fluoride-acclimated *S. cerevisiae* exhibited increased clumping and mutations to genes associated with flocculation, pseudohyphal growth, cell surface properties, and adhesion^88^. In both prokaryotic and eukaryotic microorganisms, fluoride potentiates the effect of drugs that inhibit cell wall biosynthesis^109–111^.

### Fluoride sequestration

Another mechanism of fluoride resistance is sequestration of the anion in an insoluble form, to avert its inhibition of metabolic enzymes. The molecular mechanisms of sequestration are currently not as well understood. It has been shown that some species, including the extremely fluoride-resistant *Exiguobacterium indicum* MLN15 and *Bacillus licheniformis* absorb fluoride in electron-dense granules on the surface and in the cytoplasm, respectively, although the molecular identity of these is unknown^112,113^. Bacteria are known to biomineralize calcium in crystal lattices that can incorporate fluoride, including francolite, aragonite, dolomite, and apatite^114–118^. Based on these principles, fluoride mineralization has been engineered into some bacteria^119,120^. In addition, evidence from eukaryotes suggests that fluoride sequestration is harnessed to counteract fluoride toxicity. For example, some plants accumulate fluoride as insoluble calcium or magnesium complexes in the vacuole^121^, and fluoride-acclimated yeast exhibited far lower soluble calcium compared to total calcium, suggesting calcium’s presence in an insoluble form^88^. Thus, sequestration can be used as a fluoride mitigation strategy by biology, and, in principle, could be stimulated by the divalent metal and phosphate uptake described above.

## Recommendations for leveraging fluoride resistance

The carbon atoms in perfluorinated compounds are highly oxidized already and reductive defluorination coupled to ATP generation has not, to our knowledge, been demonstrated. The high degree of fluorination coupled with the lack of known metabolic energy provided by PFAS metabolism suggests that fluoride stress is a major constraint on sustained organofluorine biodegradation in engineered, and perhaps highly contaminated, natural systems. Likewise, fluoride tolerance is required for the synthesis of organofluorine chemicals, since fluoride ions must be supplied to bacteria for fluorination reactions. In addition to host tolerance, multiple enzymes, some of which use redox and other cofactors, must operate in conditions of fluoride stress. Intersecting approaches will be needed to overcome this problem, such as using or engineering fluoride-resistant bacterial strains, protein engineering to circumvent fluoride target sensitivity, or optimizing growth conditions to help mitigate fluoride stress. We expand on some of these ideas below.

### Using naturally resistant cellular hosts

For some bacteria, like *E. coli*, fluoride accumulation is bacteriostatic, halting metabolism while fluoride is present^71^. Other bacteria, like *Bacillus subtilis*, *Neisseria subflava*, and *Streptococcus* species exhibit varying degrees of autolysis in response to fluoride stress^106^. Extreme fluoride challenges can cause complete lysis of the population^68^. Such species are obviously less suited for applications that require high fluoride levels. In contrast, some bacteria exhibit extreme fluoride tolerance, managing growth and metabolism even at high environmental fluoride concentrations. Such strains have been identified in the context of organofluorine biosynthesis^21^, biodegradation^63^ and fluoride sequestration^113^.

### Evolving or acclimating host cells to resist fluoride

Because so many fluoride resistance mechanisms involve rewiring metabolic pathways to circumvent inhibition of glycolysis, cytoplasmic acidification, or oxidative stress, adaptive evolution or fluoride acclimation has the potential to rapidly generate strains with better fluoride tolerance. We are not aware of any systematic investigation of fluoride acclimation in bacteria, but the example of *S. cerevisiae* is encouraging, as fluoride acclimation over multiple generations yielded strains with ~700-fold better fluoride resistance, independent of fluoride exporter expression^88^. However, it should be noted that one natural mechanism of fluoride resistance is metabolic deactivation, which may be at odds with the end goal of improving fluoride resistance in order to metabolize fluorinated substrates.

Thus, a more relevant application of adaptive evolution might be to drive improved fluoride stress management and defluorination rates simultaneously^35,63^. Recent studies show that using fluorinated compounds as the sole carbon source generates dual and opposite selective pressures for sufficient carbon metabolism to provide cellular metabolites and ATP, counterbalanced by toxicity from excessive intracellular fluoride flux^35,63^. This combination of metabolic needs pitted against the requirement to handle fluoride stress is an ideal problem to solve by adaptive evolution. In a comparable case, a *Pseudomonas* strain was adapted to metabolize high levels of cytotoxic hydroxycinnamic acids from lignin breakdown^122^. Adaptive evolution allows natural selection to solve the problem of high internal fluoride levels, and these strains may find utility for both PFAS biodegradation and biosynthesis.

### Engineering fluoride resistance

By engineering host bacteria, fluoride resistance could be further improved. Most straightforwardly, genes that are critical to the fluoride stress response, such as fluoride exporters, could be constitutively expressed. During PFAS biodegradation, the release of fluoride anion intracellularly can cause cessation of energy metabolism before a fluoride stress response is mounted^63^. Constitutive expression of fluoride exporters is one naturally evolved response that improves bacterial fluoride tolerance^91^. It is also possible that the expression of multiple fluoride exporters could help improve fluoride resistance. Each of the two mechanistically distinct fluoride export proteins has its own advantages, at least in principle. The F^−^/H^+^ antiport mechanism couples fluoride export to the proton gradient, sustaining a lower intracellular fluoride concentration at equilibrium, whereas fluoride channels have the advantage of more rapid fluoride removal that does not depend on a proton gradient. However, it is at least theoretically possible that at high external fluoride, if a cell is unable to maintain a membrane potential, a fluoride channel could permit fluoride influx. Protein-level regulation could prevent this outcome. Although synthetic proteins that inhibit fluoride channels have been developed^123–125^, they have not been tested in biological systems, and no such natural regulatory mechanism has been identified. Fluc channels are more widely distributed among diverse bacteria than CLC^F^ transporters (Fig. 3), and Flucs are found more commonly in strains that resist high fluoride or that use fluoride for synthesis. These observations perhaps imply that channels are biology’s favored solution to fluoride export.

We can also follow the example of nature and simply overexpress enzymes like enolase that represent key roadblocks in metabolism, those that respond to oxidative stress, or that, like pyrophosphatase, have homologs that are less sensitive to fluoride inhibition^126^. For bioremediation, the introduction of genes that contribute to external sequestration may be protective in static natural or engineered bioremediation systems in which exported fluoride might accumulate and reenter cells. One recent study showed that calcium carbonate precipitate generated by *Pseudomonas* sp. HXF1 could sequester fluoride in the form of CaF_2_ and Ca_5_(PO_4_)_3_F and diminish fluoride in groundwater^127^.

### Growth conditions that reduce fluoride stress

The natural mechanisms that microbes use to withstand fluoride toxicity suggest several straightforward ways to optimize growth conditions, including maintaining the medium at neutral pH, providing phosphate and divalent cations in the medium, or supplementation with antioxidant compounds like sulfites. Accumulation of fluoride released by PFAS degradation might prove particularly acute in, for example, an engineered bioreactor designed to degrade concentrated PFAS waste. In this situation, fluoride toxicity could be ameliorated by including a solid-phase adsorbent material to sequester fluoride following its export into the extracellular space. A variety of fluoride-binding materials have been developed for treating potable waters that naturally contain high levels of fluoride^128^. Examples include bone char, activated carbon, activated carbon with metals, and more advanced ceramic materials containing rare earth metals.

For biosynthetic applications, it may prove possible to supply bound fluoride in a slow-release form to maintain a sub-toxic, steady-state level of fluoride. One example would be the amendment of biocatalytic reaction mixtures with fluorophosphate, which could be released by phosphatases^129^. However, there is a measurable background rate of non-enzymatic fluorophosphate hydrolysis and so more stable, but inexpensive, fluoride salts like BF_4_^−^ could be efficacious in this regard.

### Integrating fluorobiosynthesis with fluoride tolerance

Finally, fluorobiosynthesis pathways themselves could be engineered to reduce the cellular fluoride burden. Similar to organisms that naturally produce organofluorine compounds^72^, temporal separation of metabolism and biosynthesis could be used in engineered systems. Alternatively, fluoride-inhibited energy metabolism could be circumvented using bacteria capable of accessing electrons from electrodes, a process particularly useful to drive reductive defluorination^130^. In addition, the discovery, characterization, and engineering of new fluorinase enzymes should focus on the discovery of enzymes with lower *K*_m_ values to reduce the intracellular fluoride required for synthesis. Orthogonal improvements to organofluorine biosynthesis pathways, like SAM regenerating systems, could also reduce the metabolic burden of fluorination chemistry, permitting high fluxes under conditions of low metabolic throughput. A SAM regenerating system has been developed for the purpose of supporting in vitro cobalamin biosynthesis and this could be useful in fluorinase biochemistry, too^131^.

## Summary and outlook

PFAS accumulation in the environment is an expanding societal problem, and microbial bioengineering shows promise for PFAS remediation or synthesis of less-fluorinated and more biodegradable chemicals to replace undesirable PFAS. Much attention within this field has been directed towards the discovery or engineering of enzymes that can break the famously strong C–F bond. Progress along this front is promising: although such enzymes are rare, recent studies show that they are more diverse than previously thought^51^, and advancements in metagenomic sequencing and protein engineering will support future discovery and optimization of organisms, genes, and pathways that support organofluorine synthesis and degradation. For example, homologs to a newly discovered reductive defluorinating enzyme system were recently identified in metagenomes found on six continents, greatly expanding the range of enzymes of this type to be studied^51^. Furthermore, we argue here that defluorination chemistry is only a part of the challenge in this field. By acquiring a deep understanding of the fundamental microbial physiologies — in particular the fluoride stress responses — that support biodegradation or biosynthesis of organofluorine molecules, we can better harness ancient fluoride resistance mechanisms to address this very contemporary biochemical problem. Box 1 describes targeted areas of research that will further advance these fields.

Box 1 AREAS FOR FUTURE RESEARCH

- Continued discovery and engineering of enzymes and metabolic pathways capable of efficient fluorination or defluorination.
- Identify mechanisms of fluoride stress management that govern cell metabolism and viability in diverse bacterial hosts using multi-omics approaches.
- Optimize regulation of fluoride tolerance mechanisms, including regulation of fluoride export activity.
- Differentiate fluoride channels versus antiporters for high-level fluoride resistance under different metabolic circumstances.
- Continued discovery and improvement of enzymes that reduce fluoride stress or that are insensitive to fluoride inhibition.
